# Single-incision laparoscopic transabdominal preperitoneal hernioplasty: 1,054 procedures and experience

**DOI:** 10.1007/s10029-023-02803-1

**Published:** 2023-05-28

**Authors:** Jingyi Jiao, Xiaojun Zhu, Chun Zhou, Peng Wang

**Affiliations:** 1 Nantong University Medical School, Nantong, 226001 China; 2 Department of Hepatobiliary and Pancreatic Surgery, Affiliated Hospital of Nantong University, No. 20 Xisi Road, Nantong, 226001 Jiangsu China; 3 Department of General Practitioner, Affiliated Hospital of Nantong University, No. 20 Xisi Road, Nantong, 226001 Jiangsu China

**Keywords:** Single-incision laparoscopic surgery, Inguinal hernia, Groin hernia, Hernioplasty, TAPP

## Abstract

**Purpose:**

Although there have been numerous studies on single-incision laparoscopic inguinal hernia repair (SIL-IHR), the short- and long-term outcomes in patients from a large single institution who underwent single-incision laparoscopic transabdominal preperitoneal hernioplasty (SIL-TAPP) have rarely been reported. The purpose of this study is to evaluate the short- and long-term outcomes of SIL-TAPP and its safety and feasibility in patients from a large single institution.

**Methods:**

The details of 1,054 procedures in 966 patients who underwent SIL-TAPP at the Affiliated Hospital of Nantong University from January 2015 to October 2022 were retrospectively analysed. SIL-TAPP was performed completely through the umbilicus using conventional laparoscopic instruments. Short-term and long-term outcomes of SIL-TAPP were collected by outpatient and telephone follow-ups. In addition, we further compared the operation time, length of postoperative hospital stay, and postoperative complications of patients with simple and complicated unilateral inguinal hernias.

**Results:**

A total of 1,054 procedures were performed for 878 unilateral inguinal hernias and 88 bilateral inguinal hernias. In total, there were 803 (76.2%) indirect inguinal hernias, 192 (18.2%) direct inguinal hernias, 51 (4.8%) femoral hernias and 8 (0.8%) combined hernias. The mean operative time was 35.5 ± 17.0 min for unilateral inguinal hernias and 51.9 ± 25.5 min for bilateral inguinal hernias. There was one (0.1%) conversion to two-incision laparoscopic transabdominal preperitoneal hernioplasty. No intraoperative haemorrhages, inferior epigastric vessel injury or nerve damage occurred. Postoperative complications were minor and could be resolved without surgical intervention. The mean length of hospital stay was 1.3 ± 0.8 days. The median follow-up was 44 months, no trocar hernia occurred, and there was one (0.1%) recurrence. The operation time in the complicated inguinal hernia group was significantly higher than that in the simple inguinal hernia group (38.9 ± 22.3 vs. 35.0 ± 15.6, *p* = 0.025). The length of postoperative hospital stay and complication rate of the complicated inguinal hernia group were slightly higher than those of the simple inguinal hernia group, but the difference was not statistically significant.

**Conclusion:**

SIL-TAPP is safe and technically feasible, and both short- and long-term outcomes are acceptable.

## Introduction

Tension-free hernia repair has become the preferred surgical procedure for inguinal hernia repair [1]. With the advent and development of laparoscopic inguinal hernia repair, the postoperative pain of patients has been greatly reduced, and the postoperative recovery speed has been greatly accelerated [2, 3]. Most importantly, laparoscopic inguinal hernia repair makes a posterior approach possible. The advantages of the posterior approach include the ability to identify the inguinal hernia more accurately and place the larger mesh in a more stable position [4].

To further reduce the invasiveness of inguinal hernia repair, SIL-IHR was developed. SIL-IHR generally refers to SIL-TAPP and single-incision laparoscopic total extraperitoneal hernia repair (SIL-TEP). Compared with conventional laparoscopic inguinal hernia repair, SIL-IHR has the potential benefits of faster postoperative recovery, less pain, and better cosmetic outcomes for patients while maintaining the advantages of a posterior approach. Due to these potential advantages, there have been numerous reports about SIL-IHR to date, suggesting that it has similar short- and long-term outcomes to conventional multi-incision laparoscopic inguinal hernia repair [4–6]. However, most reported cases were very limited in number [7] and focused on SIL-TEP, while short- and long-term outcomes of SIL-TAPP at large single institutions were rarely reported. In this study, a large number of SIL-TAPP case data from a single institution were analysed to determine the safety and feasibility of SIL-TAPP and to evaluate the short- and long-term outcomes.

## Materials and methods

### Patients

We retrospectively analysed data from the hernia disease registration and follow-up system of the Affiliated Hospital of Nantong University, which prospectively collected the detailed information of 1,285 consecutive inguinal hernia patients treated by SIL-TAPP under general anaesthesia at the Affiliated Hospital of Nantong University from January 2015 to October 2022. All patients were fully aware of the possible advantages and limitations of SIL-TAPP, conventional multi-incision laparoscopic inguinal hernia repair and open inguinal hernia repair before surgery. According to the wishes of the patients and the evaluation of the surgeon, SIL-TAPP was chosen for the patients. The exclusion criteria were as follows: (1) younger than 14 years of age; (2) acute incarcerated or strangulated hernia; (3) cardiopulmonary insufficiency; and (4) combined operations on other organs of the body. According to the strict inclusion and exclusion criteria, 966 patients meeting the requirements were finally recruited to participate in this study. All SIL-TAPPs included in this study were completed by the same experienced surgeon and multiple assistants, and all were completed with the informed consent of the patients.

### Surgical techniques

**Fig. 1:**
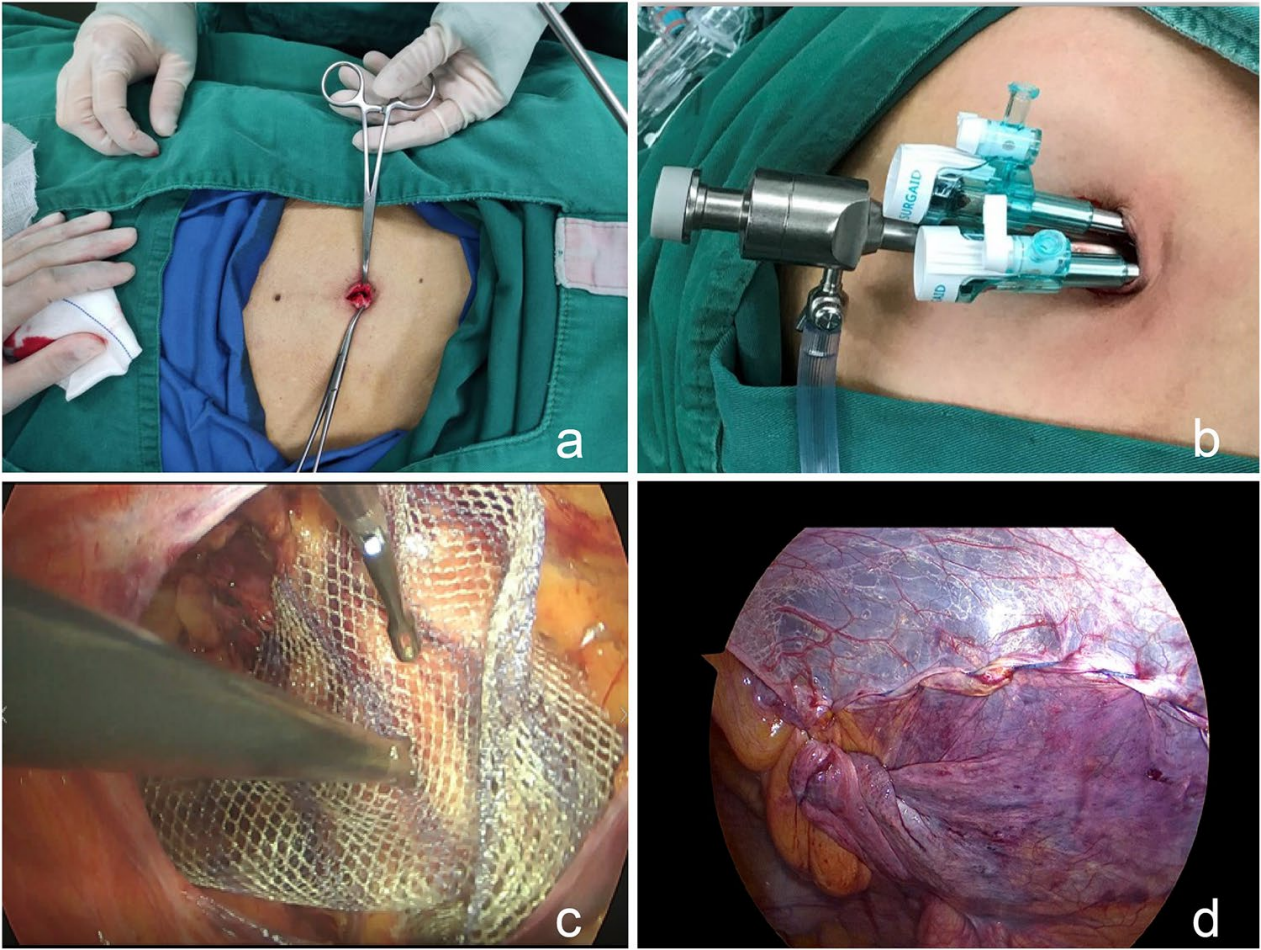
Photographs of some details of SIL-TAPP. **a** A transumbilical incision was made, and the umbilical ring was opened. **b** One 10 mm trocar and two 5 mm trocars were inserted by puncture. **c** The mesh was placed and unfolded fully. **d** The peritoneum was sutured continuously

All patients had their navel cleaned and the hair in the surgical area shaved before the operation, and anaesthesia was a combination of intravenous injection and inhalation. After anaesthesia was successful, the belly button was disinfected again with alcohol cotton balls. A longitudinal incision of approximately 20 mm was made through the umbilicus, blunt free subcutaneous tissue to open the umbilical ring, and the umbilical flap was turned outwards to establish the “fascia platform” (Fig. 1a). The 10 mm trocar was inserted into the umbilical “fascia platform” under direct vision and a CO_2_ pneumoperitoneum was then established with the pressure set at 12-15 mmHg. Two 5 mm trocars were inserted into the umbilical fascia platform as operating holes (Fig. 1b), with separation forceps and electrical hooks inserted. Laparoscopy was performed to explore the abdominal cavity and pelvic cavity to determine the position of the hernia sac. Then, the patient was placed in the reverse Trendelenburg position with the hernia site rotated upwards. At the level of 3 cm along the upper margin of the inner ring opening, the peritoneum was cut from the medial umbilical fold to the anterior superior iliac spine. When crossing the lateral umbilical fold, the peritoneum was pulled by separation forceps to allow CO_2_ gas to enter the preperitoneal space and assist in separating the inferior epigastric vessel from the peritoneum to avoid vessel damage. The upper and lower margins of the peritoneal flap were dissociated, and the preperitoneal space was entered. The Bogros space of the lateral margin was dissociated to the middle of the iliopsoas muscle and then to the lateral margin of the hernia sac. Protection of the transversalis fascia was considered throughout the process to avoid damage to the lateral femoral cutaneous nerve and the femoral branch of the genitofemoral nerve in the pain triangle. The transversalis fascia and bladder were separated from the medial side of the inferior epigastric vessel to the central side, entering the Retzius space, exposing from the pectineal ligament to the pubic symphysis, expanding inwards across the midline, and dissociating outwards to the medial margin of the hernia sac. Protecting the bladder and the corona mortis across the pectineal ligament was given consideration. The direct hernia sac was completely returned, and the fixation of the “false hernia sac” was determined according to the situation. For hernia sac with a diameter of more than 2.5 cm, the “false hernia sac” was pulled back and fixed to the pectineal ligament. For an indirect hernia, the noninvasive forceps in the left hand were used to pull the hernia sac or peritoneum and maintain tension, while the electric separation forceps in the right hand were used to pull the hernia sac, identify the space between the peritoneum and the genital vessels/seminiferous duct, and separate alternately on both sides of the hernia sac. If it could be separated to the apex, the hernia sac was completely detached. If the hernia sac was large and densely adhered to the surrounding tissue, it was transected, and the distal end was opened. The hernia sac needs to be separated medially beyond the midline, and the bilateral hernia needs to be connected on both sides, laterally to the level of the anterior superior iliac spine, superiorly to about 2–3 cm above the conjoined tendon, medially inferiorly to about 2 cm below the pectineal ligament, and laterally inferiorly to the level of the middle iliopsoas muscle (approximately 6 cm below the opening of the internal ring). A mesh of approximately 10 cm × 15 cm was selected to implant into the preperitoneal space to cover the separation area above (Fig. 1c). Peritoneal incision suturing is the most difficult step for SIL-TAPP. The absence of trigonal relationships and the “chopstick effect” between instruments usually lead to a significant prolongation of peritoneal suture time and operative time under the endoscope, which is the main reason for the long learning curve of SIL-TAPP. The continuous suture is the most suitable peritoneal closure; although it is more time-consuming, the patient has less postoperative pain. It is recommended to use a 4-0 v-lock to suture the peritoneum continuously from lateral to medial without knotting (Fig. 1d). With this method, the difficulty of suturing is less, and the suture time and the surgeon’s learning curve are shorter. Peritoneal suturing should be performed gently to avoid damage to the abdominal organs. Finally, several stitches were sutured on the thick tissue. After the suture was completed, the suture was cut without retaining the residual end of the suture to avoid intestinal injury and intestinal obstruction. Reducing the intraperitoneal pressure (8 mmHg or lower) often facilitates continuous closure of the peritoneum, especially in difficult cases. If the peritoneal tension was large, titanium clamps were used to clamp the residual end of the suture. The one-hand peritoneal suture technique was used to mitigate the “chopstick effect”. Once the suture was deemed satisfactory, the pneumoperitoneum was drained under direct vision to ensure that the repair was complete. For the midline longitudinal incision of the umbilicus, the suture was usually divided into two layers. First, the umbilical fascia layer was sutured continuously with a 1-0 v-lock suture, and then the midpoint of the standing flap at the umbilical ring was sutured with an absorbable protein line. Both ends were sutured intradermally, and the rest were sutured intermittently. To reduce skin tension, the subcutaneous tissue was trimmed. After the bleeding was cleaned with gauze, the standing flap was pushed back to the umbilical fossa to achieve a perfect repair.

### Follow-up

All patients in the hernia disease registration and follow-up system were followed up 1 week after surgery to assess short-term complications, including wound pain, infection, seroma, and haematoma. In addition, patients were followed up by telephone at 1, 3, and 6 months after surgery and every 6 months thereafter to assess chronic pain, trocar hernia, recurrence, and other long-term complications.

### Statistical analysis

All analyses were performed with SPSS version 26 (IBM). The data are given as the mean ± SD, number, or number (%). A *p* value of < 0.05 was considered statistically significant.

## Results

The characteristics of the patients and their inguinal hernias are summarized in Table 1. A total of 1,054 inguinal hernias were repaired in 966 patients during the study period (unilateral inguinal hernias in 878 cases and bilateral inguinal hernias in 88 cases). Of the 966 patients, 840 were male and 126 were female. The mean age of patients was 58.0 ± 17.8 years, with the minimum age of patients being 15 years and the maximum age is 93 years. The average body mass index of the patients was 23.1 ± 3.4 kg/m^2^. The median follow-up time was 44 months, and the follow-up rate was 99.3%; 7 patients died from other causes. Four types of hernia were found during surgery: indirect inguinal hernia, direct inguinal hernia, femoral hernia, and combined hernia. A combined hernia is defined as any combination of the other 3 types of inguinal hernia. Of these, the indirect inguinal hernia was the most common type (76.2%).

**Table 1:** Patient and hernia characteristics (966 patients and 1,054 hernias)

Variable	Value
Number of patients	966
Sex	
Male	840
Female	126
Age (years)	58.0 ± 17.8
Body mass index (kg/m^2^)	23.1 ± 3.4
Site of hernias	
Left	382 (39.5%)
Right	496 (51.3%)
Both	88 (9.1%)
Type of hernias	
Indirect inguinal hernias	803 (76.2%)
Direct inguinal hernias	192 (18.2%)
Femoral hernias	51 (4.8%)
Combined inguinal hernias	8 (0.8%)

Values are reported as numbers and percentages unless otherwise indicated

As shown in Table 2, the average operation time for unilateral inguinal hernia was 35.5 ± 17.0 min and that for bilateral inguinal hernia was 51.9 ± 25.5 min. One case (0.1%) was converted to two-incision laparoscopic inguinal hernia repair because the patient was a recurrent inguinal hernia patient with serious adhesion in the inguinal region, and the separation angle of the single-incision laparoscopic instrument was not sufficient. Mean intraoperative blood loss was minimal, and no inferior epigastric vessel injury or massive bleeding occurred. The incidence of postoperative complications was 18.5%. Among them, 1 patient had a recurrence (0.1%). None of the patients developed trocar hernias. One patient developed inguinal haematoma (0.1%). Inguinal seroma was observed in 156 patients (14.8%). Umbilical cord infection occurred in 5 patients (0.5%). Chronic groin pain occurred in 9 patients (0.9%). No mesh infection occurred. All postoperative complications were resolved without surgical intervention. Five patients (0.5%) with infected superficial umbilical incisions were cured by disinfection and dressing changes at the outpatient clinic. All urinary retention occurred shortly after surgery, and patients did not have long-term bladder dysfunction. All patients with postoperative seroma and haematoma recovered after conservative treatment or percutaneous aspiration.

**Table 2:** Operative results (966 patients and 1,054 hernias)

Variable	Value
Operation time (min)	
Unilateral hernias	35.5 ± 17.0
Bilateral hernias	51.9 ± 25.5
Conversion to conventional laparoscopic surgery	1 (0.1%)
Blood loss (ml)	Minimal
Postoperative hospital stay (days)	1.3 ± 0.8
Intraoperative complication	
Major bleeding	0
Vascular injury	0
Nerve injury	0
Postoperative complication	
Seroma	156 (14.8%)
Hematoma	1 (0.1%)
Urinary retention	22 (1.1%)
Wound infection	5 (0.5%)
Trocar hernias	0 (0.0%)
Recurrence	1 (0.1%)
Chronic pain	9 (0.9%)
Mesh infection	0 (0.0%)

Values are reported as numbers and percentages unless otherwise indicated

To determine whether there was a difference in outcomes between simple and complicated unilateral inguinal hernia surgery with SIL-TAPP, we further compared operation time, length of postoperative hospital stay, and postoperative complications between simple and complicated unilateral inguinal hernia surgeries. The complicated inguinal hernia was defined as (1) recurrent inguinal hernia; (2) inguinal hernia after previous major surgery in the lower abdomen; and (3) nonnecrotizing incarcerated inguinal hernia in which the hernia contents were omental or bowel. Table 3 includes operation times, lengths of postoperative hospital stay, and postoperative complications for simple and complicated unilateral inguinal hernias. The operation time of a complicated unilateral inguinal hernia was significantly longer than that of a simple inguinal hernia. The length of postoperative hospital stay and complications in patients with complicated unilateral inguinal hernia were greater than those in patients with simple inguinal hernia, but there was no statistical significance.

**Table 3:** Comparisons between unilateral simple and complicated inguinal hernias

	Simple inguinal hernias (*n* = 773)	Complicated inguinal hernias (*n* = 105)	*p* value
Operation time (min)	35.0 ± 15.6	38.9 ± 22.3	0.025
Postoperative hospital stay (day)	1.3 ± 0.4	1.3 ± 0.7	0.205
Postoperative complications	154 (19.9%)	25 (23.8%)	0.354

Values are reported as numbers and percentages unless otherwise indicated

## Discussion

Inguinal hernia is a common clinical disease, with a lifetime incidence of 27% in males and 3% in females [8]. Surgical repair is the most effective treatment. It is estimated that more than 20 million inguinal hernia repairs are performed globally each year, making it one of the most common surgical procedures in the world [9]. Inguinal hernia surgery includes open surgery and laparoscopic surgery. At present, laparoscopic inguinal hernia repair is the best way to treat inguinal hernia, replacing open inguinal hernia repair. The advantage of laparoscopic surgery for inguinal hernia is that the hernia can be reduced and repaired under direct line of sight, thus making the procedure more accurate.

To further reduce the invasiveness of inguinal hernia repair, SIL-IHR was developed. Since Dr Cugura performed the first SIL-IHR in 2008, the technique has become more widely accepted [10]. The term SIL-IHR generally refers to SIL-TAPP and SIL-TEP. Compared to conventional multi-incision laparoscopic inguinal hernia repair and open inguinal hernia repair, SIL-IHR offers unique advantages, including shorter recovery periods, better cosmetic outcomes, and similarly low or improved recurrence and complication rates. Based on our experience with over 1,000 SIL-TAPP surgeries and numerous detailed studies, SIL-TAPP may have advantages over all other laparoscopic or open inguinal hernia repairs.

### Short operation time

According to previous reports, due to the “chopstick effect” caused by the single incision and the absence of the “operating triangle”, single-incision laparoscopic inguinal hernia repair, whether SIL-TAPP or SIL-TEP, can lead to a significant increase in surgical time, which may lead to increased surgical risk [11, 12]. However, in our study, the mean operating time was 35.5 ± 17.0 min for unilateral inguinal hernias and 51.9 ± 25.5 min for bilateral inguinal hernias. Among them, the operation time of one male direct inguinal hernia patient was approximately 8 min, and the operation time of one female indirect inguinal hernia patient was approximately 10 min. Our surgical time was much lower than that previously reported for SIL-TAPP and even lower than the average surgical time for conventional multi-incision laparoscopic inguinal hernia repair [13]. Because the suturing of the fascia layer and skin layer of the belly button was performed by different, less experienced assistants under the guidance of the surgeon, the overall surgical time was extended during the belly button suturing stage, and the actual surgical time was shorter than that shown in Table 2.

In addition, due to the short operation time, none of our patients in this study had indwelling catheters before surgery, which greatly reduced patients’ discomfort and improved their satisfaction with surgery. Before the study began, our team had completed more than 300 single-incision laparoscopic cholecystectomies (SIL-LC) and single-incision laparoscopic appendectomies (SIL-LA), and the surgeon had already mastered the single-incision laparoscopic technique. Therefore, we believe that after passing the SIL-TAPP learning curve, the surgical time of SIL-TAPP is not significantly different from that of conventional multi-incision laparoscopic inguinal hernia repair or could be even lower, so there is no increase in related surgical risks.

### Wide range of indications

At present, single-incision laparoscopy is mainly used in cholecystectomy, appendectomy, colorectal resection, myomectomy and inguinal hernia repair [4, 14–17]. As a result, some people believe that single-incision laparoscopic surgery can only be performed in relatively simple operations, and SIL-TAPP can only be applied for relatively simple inguinal hernias. For some difficult and complex inguinal hernias, conventional multi-incision laparoscopic inguinal hernia repair and open inguinal hernia repair are still the preferred operations.

However, our study included many very complicated and difficult inguinal hernia patients, such as inguinal hernia patients after robotic prostatic surgery, inguinal hernia patients after three open uterine surgeries, recurrent inguinal hernia patients with cirrhosis after bilateral prostatic surgery, and giant hernia patients with cirrhosis ascites. The anatomical relationships of these patients are so complex that even conventional multi-incision laparoscopic inguinal hernia repair and open inguinal hernia repair are difficult to manage. With our efforts, we still completed SIL-TAPP for our patients, and these patients recovered quickly after surgery. The results in Table 3 also indicate that there was no significant difference in the outcomes of patients with complicated inguinal hernia and simple inguinal hernia treated with SIL-TAPP surgery, except for the longer operation time. Additionally, SIL-TAPP makes contralateral occult inguinal hernias easier to detect, and preventive repair of contralateral occult inguinal hernias may prevent the need for repeat surgery in nearly a third of patients [18]. SIL-TAPP is also convenient for the observation and treatment of incarcerated hernias and hernia contents. In addition to this study, we successfully performed many SIL-TAPP procedures combined with SIL-LC, SIL-LA, and single-incision laparoscopic resection of round ligament cysts, and the patients recovered well after surgery. These results suggest that SIL-TAPP is not only suitable for simple inguinal hernias but also a reliable choice for more difficult and complex inguinal hernias.

### No inferior epigastric vessel injury

The inferior epigastric vessel includes the inferior epigastric artery and inferior epigastric vein, and the body surface projection shows the midpoint of the inguinal ligament moving towards the umbilical cord. The main symptoms of inferior epigastric vessel injury are local pain, bleeding, and haematoma formation or shock. Haematoma dilatation, subcutaneous congestion, and stasis can also compress adjacent nerves, leading to local numbness and paraesthesia. Conventional multi-incision laparoscopic inguinal hernia repair involves a risk of damage to the inferior epigastric vessel during bilateral trocar puncture. In a retrospective study of 3,100 patients undergoing conventional multi-incision laparoscopic tension-free inguinal hernia repair, the inferior epigastric vessel injury rate was 0.47% [19]. Avoiding blind puncture of the bilateral trocar is the key to reducing damage to the inferior epigastric vessel. Therefore, a longitudinal incision of approximately 20 mm was made in the umbilical cord into the abdominal cavity for SIL-TAPP. There was no blind penetration, so damage to the inferior epigastric vessel could be avoided during bilateral trocar puncture. In our study, there was no inferior epigastric vessel injury. This suggests that transumbilical SIL-TAPP has advantages over conventional multi-incision laparoscopic inguinal hernia repair in avoiding inferior epigastric vessel injury.

### The incision is small and concealed

**Fig. 2:**
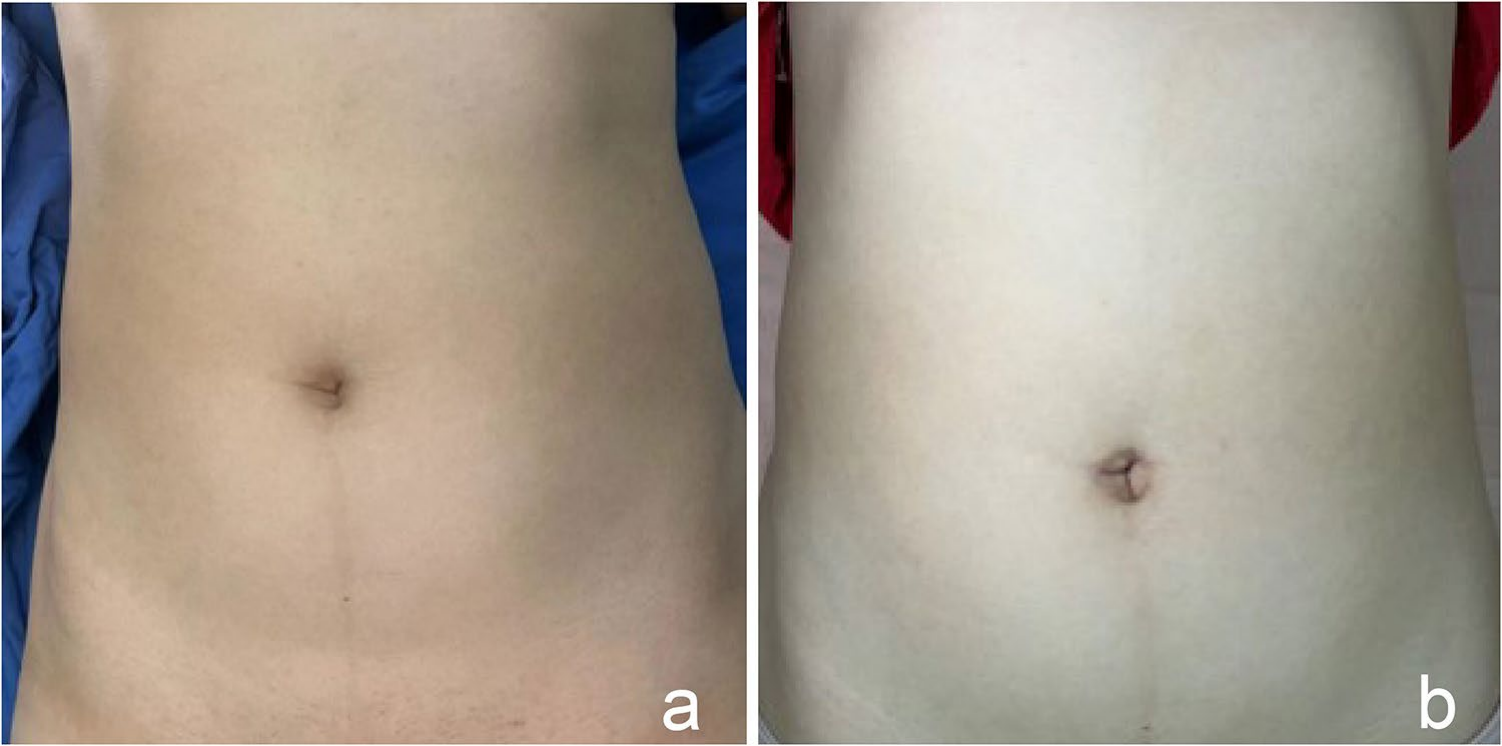
Belly button before and after SIL-TAPP. **a** Belly button before SIL-TAPP. **b** Belly button after SIL-TAPP

The elimination of inguinal symptoms is no longer the only criterion for measuring the success of inguinal hernia surgery. Cosmetic effects are also important indicators for patients to consider when choosing surgical procedures [20]. A remarkable cosmetic effect is the unique advantage of single-incision laparoscopic surgery, and small and hidden incisions are the original intention of SIL-TAPP for most inguinal hernia patients. At the beginning of the operation, an incision of approximately 20 mm was made longitudinally in the umbilicus to open the umbilical ring and turn the umbilicus flap outwards to establish a “fascia platform”. In this way, the umbilical fossa still has enough depth to accommodate the standing flap after the fascia layer suture, and the postoperative umbilical cord of patients is more natural and aesthetically pleasing. In addition, unlike most previously reported studies, all SIL-TAPPs included in this study involved the insertion of the trocar entirely through the umbilicus rather than the subumbilicus or other locations. This trocar placement method integrates the postoperative scar and umbilical fossa fold, resulting in a relatively hidden, almost invisible postoperative scar and high patient satisfaction (Fig. 2a, b).

### No trocar hernia occurred

Trocar hernia is a serious complication after laparoscopic surgery, often presenting with nausea, vomiting, abdominal pain, poor exhaust and bowel movement, and abdominal incision mass or bulge. In previous reports, patients undergoing single-incision laparoscopic surgery had an increased incidence of trocar hernia compared with conventional multi-incision laparoscopic surgery [21]. The most important factor leading to trocar hernia is improper suturing, and fascial layer suturing is very important in preventing trocar hernia. Before the insertion of the trocar by transumbilical puncture, an incision of approximately 20 mm was made longitudinally in the umbilical cord, and the umbilical ring was opened to facilitate the suture of the final fascia layer. For longitudinal umbilical incisions, we usually use two layers of the suture. First, the umbilical fascia layer was sutured continuously with a 1-0 v-lock suture. Then, the midpoint of the standing flap at the umbilical ring was sutured with an absorbable protein suture, both ends were sutured intradermally, and the rest were sutured intermittently. To reduce skin tension, the subcutaneous tissue was trimmed. A gauze was used to remove the residual blood from the umbilical fossa, and the standing flap was pushed back to the umbilical fossa to achieve the perfect repair. After the wound healed, the scar was almost invisible. During the telephone follow-up, to know whether the patient developed an umbilical trocar hernia after surgery, we required the patient to truthfully answer the following questions: (1) whether the umbilical wound exhibited redness, swelling or exudation; (2) whether there was an obvious bulge in the umbilicus; and (3) whether any redness, swelling or exudation had recovered and how long recovery took. If the symptoms did not resolve, the patient was requested to come to the hospital for a further examination to determine the nature of the bulge by palpation and ultrasound. In addition, we kept in close contact with patients through WeChat, and we were able to determine whether the patient had an umbilical trocar hernia by the standing position photos they sent. Follow-up results showed that no umbilical trocar hernia occurred. This indicates that this suture method is beneficial to prevent the occurrence of trocar hernia. These results also proved that there was no significant relationship between SIL-TAPP and the occurrence of trocar hernia, and SIL-TAPP did not lead to an increased incidence of trocar hernia compared with conventional multi-incision laparoscopic inguinal hernia repair. However, as stated above, not all patients underwent physical examination and ultrasound during follow-up, so there was a possibility of missing trocar hernias. We acknowledge that this is one of the limitations of our study and that further research is needed to confirm the incidence of trocar hernias.

### Limitations of SIL-TAPP

The surgeons in our team had performed more than 300 single-incision laparoscopic cholecystectomies and single-incision laparoscopic appendectomies before performing SIL-TAPP surgery, accumulating rich experience in single-incision laparoscopic surgery, so that they could perform SIL-TAPP surgery safely and quickly. However, for a surgeon without experience in single-incision laparoscopic surgery, SIL-TAPP surgery is difficult, and the safety of surgery cannot be guaranteed. Currently, there is little literature describing the learning curve of SIL-TAPP surgery. However, recent studies have shown that experienced laparoscopic surgeons require approximately 60 to 85 SIL-TEP operations to overcome the SIL-TEP learning curve and achieve proficiency [22]. We believe that it may take more effort for beginners to overcome the learning curve of the SIL-TAPP procedure. In addition, the SIL-TAPP procedure may be difficult to complete in some particularly complex inguinal hernia patients. Therefore, SIL-TAPP surgery may never completely replace conventional multi-incision laparoscopic surgery. However, the SIL-TAPP operation gives patients another choice and allows them to choose the surgery that is suitable for them according to their expectations, which we think is a kind of medical progress.

## Conclusion

SIL-TAPP is a safe and feasible method for inguinal hernia repair that is comparable to conventional multi-incision laparoscopic inguinal hernia repair and open inguinal hernia repair. In the 1990s, laparoscopic inguinal hernia repair gradually replaced open inguinal hernia repair as the new preferred procedure. We believe that SIL-TAPP will become a routine option for inguinal hernia patients in the near future with its unique advantages.

## Data Availability

Data from this study will not be shared to protect the identity of patients in this study.

## References

[1] Simons MP, Smietanski M, Bonjer HJ (2018). International guidelines for groin hernia management. Hernia.

[2] Harmankaya S, Öberg S, Rosenberg J (2022). Varying convalescence recommendations after inguinal hernia repair: a systematic scoping review. Hernia.

[3] Aiolfi A, Cavalli M, Ferraro SD (2021). Treatment of inguinal hernia: systematic review and updated network meta-analysis of randomized controlled trials. Ann Surg.

[4] Lee YJ, Kim JH, Kim CH, Lee GR, Lee YS, Kim HJ (2021). Single incision laparoscopic totally extraperitoneal hernioplasty: lessons learned from 1,231 procedures. Ann Surg Treat Res.

[5] Wakasugi M, Hasegawa J, Ikeda Y (2021). Single-incision laparoscopic totally extraperitoneal inguinal hernia repair with tumescent local anesthesia: report of more than 2000 procedures at a day-surgery clinic. Surg Today.

[6] Chen Q-L, Chen K, Huang D-Y (2020). Trans-umbilical single-incision laparoscopic trans-abdominal pre-peritoneal hernioplasty of inguinal hernia by self-made glove port. Medicine (Baltimore).

[7] Perivoliotis K, Tzovaras G, Sarakatsianou C, Baloyiannis I (2019). Current status of single-port versus multi-port approach in laparoscopic inguinal hernia mesh repair: an up-to-date systematic review and meta-analysis. Hernia.

[8] Itani KMF, Fitzgibbons R (2019). Approach to groin hernias. JAMA Surg.

[9] Kingsnorth A, LeBlanc K (2003). Hernias: inguinal and incisional. Lancet.

[10] Filipovic-Cugura J, Kirac I, Kulis T, Jankovic J, Bekavac-Beslin M (2009). Single-incision laparoscopic surgery (SILS) for totally extraperitoneal (TEP) inguinal hernia repair: first case. Surg Endosc.

[11] Kim JH, An CH, Lee YS, Kim HY, Lee JI (2015). Single incision laparoscopic totally extraperitoneal hernioplasty (SIL-TEP): experience of 512 procedures. Hernia.

[12] Tanoue K, Okino H, Kanazawa M, Ueno K (2016). Single-incision laparoscopic transabdominal preperitoneal mesh hernioplasty: results in 182 Japanese patients. Hernia.

[13] McCormack K, Wake BL, Fraser C, Vale L, Perez J, Grant A (2005). Transabdominal pre-peritoneal (TAPP) versus totally extraperitoneal (TEP) laparoscopic techniques for inguinal hernia repair: a systematic review. Hernia.

[14] Jin HY, Lee CS, Lee YS (2021). Single incision laparoscopic appendectomy using a new multi-joint articulating instrument. J Gastrointest Surg.

[15] Fathalizadeh A, Sapci I, Gorgun IE (2021). Single-incision laparoscopic proctectomy using a magnet retractor. Dis Colon Rectum.

[16] Furukawa K, Asaoka T, Mikamori M (2022). Single-incision laparoscopic cholecystectomy: a single-centre experience of 1469 cases. J Gastrointest Surg.

[17] Einarsson JI (2010). Single-incision laparoscopic myomectomy. J Minim Invasive Gynecol.

[18] Park JB, Chong DC, Reid JL, Edwards S, Maddern GJ (2022). Should asymptomatic contralateral inguinal hernia be laparoscopically repaired in the adult population as benefits greatly outweigh risks? A systematic review and meta-analysis. Hernia.

[19] Dulucq J-L, Wintringer P, Mahajna A (2009). Laparoscopic totally extraperitoneal inguinal hernia repair: lessons learned from 3,100 hernia repairs over 15 years. Surg Endosc.

[20] Campanelli G (2022). Quality of life is the most important outcome measure of hernia repair. Hernia.

[21] Connell MB, Selvam R, Patel SV (2019). Incidence of incisional hernias following single-incision versus traditional laparoscopic surgery: a meta-analysis. Hernia.

[22] Park YY, Lee K, Oh ST, Lee J (2022). Learning curve of single-incision laparoscopic totally extraperitoneal repair (SILTEP) for inguinal hernia. Hernia.

